# Beyond facial width-to-height ratios: bizygomatic width is highly sexually dimorphic when adjusting for allometry

**DOI:** 10.1098/rsbl.2022.0211

**Published:** 2022-10-12

**Authors:** Neil R. Caton, Barnaby J. W. Dixson

**Affiliations:** ^1^ School of Psychology, The University of Queensland, Saint Lucia, Queensland, Australia; ^2^ School of Psychology, The University of the Sunshine Coast, Sunshine Coast, Queensland, Australia; ^3^ Evolution & Ecology Research Centre, School of Biological, Earth & Environmental Sciences, University of New South Wales, Sydney, New South Wales, Australia

**Keywords:** facial width-to-height ratio, bizygomatic width, sexual dimorphism, positive allometry, spurious correlation, multivariate measures

## Abstract

A large and ever-growing literature implicates male facial width-to-height ratio (bizygomatic width divided by facial height) as a secondary sexual trait linked to numerous physical and psychological perceptions. However, this research is based entirely on the premise that bizygomatic width is sexually dimorphic, which recent research has called into question. Unfortunately, statisticians for the last 125 years have noted that morphological ratio measurements may engender spurious correlations and biased effect-size estimates. In the current study, we find that bizygomatic width is highly sexually dimorphic (equivalent *d* = 1.39), even after adjusting for 92 allometric measurements, including multiple facial height and other craniofacial measurements (equivalent *d* = 1.07) in a sample of 6068 men and women. By contrast, facial width-to-height ratio (fWHR) measurements demonstrated a statistical pattern consistent with the age-old argument that morphological ratio measurements may engender spurious correlations and biased effect-size estimates. Thus, when avoiding facial ratio measurements and adjusting for allometry in craniofacial measures, we found strong support for a key premise in the human evolutionary and behavioural sciences that bizygomatic width exhibits male-biased sexual dimorphism.

## 1. Introduction

Faces epitomize complex phenotypes and communicate a myriad of social and emotional characteristics. Inspired by research among non-human animals, studies of human facial morphology propose sexual selection has shaped men's faces to communicate mate qualities, social dominance and physical formidability [1–3]. Among the multitude of facial metrics employed in face perception research, facial width-to-height ratio (fWHR) has emerged as arguably the most popular over the last 15 years [1]. fWHR is measured by dividing the distance from the left and right zygon by the distance between the upper lip and brow, putatively providing an index of mid-face robustness that is sexually dimorphic, being larger in men, on average, than women [4]. Men with larger fWHRs (i.e. wider faces) are perceived as more masculine, socially dominant and physically aggressive, but not more attractive, than men with smaller fWHRs, suggesting intra-sexual selection as the principle driving force behind sex differences in fWHR [1,2]. These perceptions reveal a kernel of truth, as larger fWHRs are positively associated with men's social status, financial success, physical formidability, number of short-term relationships and mating success [1,2].

While male fWHR is often accepted to be an androgen-dependent secondary sexual trait [1,2], meta-analyses reveal sex differences in fWHR are small (*d* = 0.11 [1]), differ depending on how fWHR was measured [2] or are absent [3]. Male fWHR is not associated with androgens during fetal development [5] or puberty [6], and polymorphisms in the androgen receptor genes in adulthood [7]. Moreover, neither baseline testosterone, nor changes in testosterone following competition, are associated with male fWHR [8]. Reconciling whether fWHR is a target of sexual selection is challenging when extant evidence does not to conform to the criteria for a sexually dimorphic secondary sexual characteristic [9].

Facial morphology is defined by complex interactions among suites of correlated traits [4]. This scaling relationship between the size of a morphological feature and overall body size is termed allometry, routinely captured using principal components analyses (PCAs) [10–25]. The resultant components of major morphological variation represent allometry, often simply termed size [10–25] and recent research has recommended that these size measurements should be modelled as covariates to adjust for allometry rather than using a ratio method [26]. It is important to account for allometric effects in facial morphology research because it otherwise remains unknown whether the morphological feature is merely a by-product of an overarching developmental system responsible for the combined growth of all morphological features [9]. Unfortunately, ratio measures such as fWHR likely capture only a small portion of allometric variation, potentially conflating positive allometric relationships among correlated traits [7,9].

While using ratios has the advantage of experimental simplicity, statisticians, including foundational theorists like Pearson, Neyman and Tanner, have for over a century criticized their use for producing spurious correlations, biased effect-size estimates [27,28], and even reducing scientific reproducibility [26]. Indeed, Karl Pearson coined the term *spurious correlation* to describe the bizarre statistics that may result from ratio measures [27], which given the lack of evidence that fWHR represents a secondary sexual characteristic suggests some reconsideration of its importance in understanding how sexual selection may have shaped men's facial morphology. While not all ratio measurements (in the broadest sense) are in themselves problematic, anatomical ratio measurements have been considered a less appropriate method of allometric scaling and may engender spurious correlations and biased effect-size estimates [26,28].

The use of ratio methods has been criticized in a related area on waist-to-hip ratio [29–33]. Inconsistencies in waist-to-hip ratio findings [33] and sexual dimorphism in fWHR [9] may be the result of using discrete ratio measures and not accounting for allometric relationships among interrelated facial and bodily traits. Thus, multivariate approaches could shed light on whether fWHR is sexually dimorphic and confirm its value as a craniofacial metric in human behavioural research.

The current study examines whether fWHR is sexually dimorphic using a uniquely population-representative dataset (see Method) from 6068 United States (U.S.) military personnel and reservists. This represents the largest and most comprehensive study to date examining sexual dimorphism in fWHR and bizygomatic width with allometric scaling (for comparison, see the meta-analysis by Geniole *et al*. [1]). In line with the recommendations of recent research [26], we accounted for allometric scaling by using bizygomatic width and controlling for 92 anatomical measurements, including facial height measurements, represented by principal components (for more detailed discussion of PCAs in allometry, see [10–25]). We then sought to confirm whether fWHR exhibits male-biased sexual dimorphism (i.e. an anatomical sex difference in which males are larger) and positive allometry. The present study represents the most comprehensive attempt to date to demonstrate whether bizygomatic width, a widely used yet controversial target of sexual selection in human males, exhibits sexual dimorphism.

## 2. Materials and methods

### (a) Data

**Figure 1.**
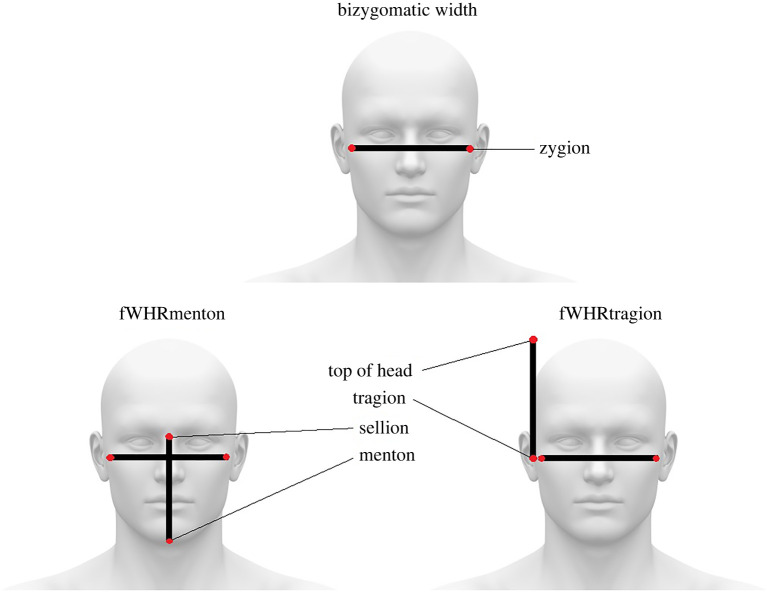
Facial measurements used in the present work.

Data were drawn from the ANSUR II dataset [34]. It includes 93 anatomical measurements (see electronic supplementary material) for 6068 adult U.S. army personnel (4082 men, *M* age = 30.16 ± 8.81; 1986 women, *M* age = 28.94 ± 8.33), including facial height measurements (e.g. menton–sellion length, tragion to top-of-head; figure 1) and more general facial measurements (e.g. jaw size, chin size and head breadth). Detailed anthropometric descriptions are included in Paquette *et al*. [34, p. 8–11], and interested readers can visit https://www.openlab.psu.edu/ansur2/ for more information. Anthropometric measurements are drawn from both military personnel and reservists from 285 unique specialties, from musicians and public affairs specialists to dental specialists and chaplain assistants, enabling the data to be close to the U.S. population [34]. For example, men's (*M* = 142.62 ± 6.22) and women's (*M* = 133.77 ± 5.56) bizygomatic width in this sample is close to the U.S. population average (average U.S. male: 140.67 ± 6.02; average U.S. female: 131.81 ± 5.09; [35]). The average sex difference was 8.85 mm in the current dataset and 8.86 mm in the U.S. population sample. Like most U.S.-based samples in facial measurements, we should note that these samples can be ethnically mixed.

Bizygomatic width was measured using spreading calipers placed on the left and right zygion landmarks, with the distance between measured in millimetres (for visual depictions of the process, see Paquette *et al*. [34, p. 32]). This is similar to placing virtual landmarks on the left and right zygion landmarks in two-dimensional photographs. All measurements in the current work were measured using callipers, tape measurements and scales (for visual depictions of each anatomical feature's measurement, see Paquette *et al*. [34]); three-dimensional body scans were also collected, but are not publicly available. We divided bizygomatic width by menton–sellion length and tragion to top-of-head to create two fWHR measurements, respectively: fWHRmenton and fWHRtragion (figure 1). We should be clear that these are new measures of fWHR, especially for fWHRtragion, with fWHRmenton most closely resembling facial width-to-lower face height (see [6,36–38]. Data are publicly available on the Open Science Framework (https://osf.io/yckrd/) [39].

### (b) Methods

All statistical symbols, methods and models are described in detail in the electronic supplementary material. Briefly, our models explicitly follow the recommendations of Nakagawa *et al*. [26]. In line with previous guidelines [10–25], size measurements were calculated using PCAs, with these major sources of allometric variation (represented by PCs) being modelled as follows: (i) as an interaction term with sex to estimate the allometric scaling component [26]; (ii) covariates in regression models to estimate size-adjusted sexual dimorphism [26]. We direct interested readers to the electronic supplementary material where we conduct and perform additional analytical considerations, as discussed in [26], which exhibit the same pattern of results.

## 3. Results

### (a) Bivariate associations between sex and bizygomatic width, fWHRmenton and fWHRtragion

There was a significant, positive bivariate association between sex and bizygomatic width (*β* = 0.57, *t* = 53.85, *p* < 0.001), indicating male-biased sexual dimorphism in bizygomatic width (equivalent *d* = 1.39). For the fWHR measurements, there were conflicting results. There was a significant, negative bivariate association between sex and fWHRmenton (*β* = −0.13, *t* = −9.95, *p* < 0.001, equivalent *d* = −0.26), whereas a significant, positive association between sex and fWHRtragion (*β* = 0.21, *t* = 17.24, *p* < 0.001, equivalent *d* = 0.43), indicating female-biased and male-biased sexual dimorphism, respectively.

### (b) Allometric scaling and bizygomatic width, fWHRmenton and fWHRtragion

Bizygomatic width exhibited positive allometry, being positively associated with 91 of the 92 anatomical measurements in men (see electronic supplementary material, table S3) with similar results for women (see electronic supplementary material, table S4). The fWHRmenton and fWHRtragion measurements demonstrated less consistent associations, as shown by its assortment of positive significant, negative significant and non-significant associations with other anatomical measurements (electronic supplementary material, tables S3 and S4).

### (c) Principal components analysis

In line with previous guidelines for calculating allometry, a PCA was run on the 92 features, excluding bizygomatic width (see electronic supplementary material). For the fWHR measurements, we also ran PCAs on the 91 features (excluding bizygomatic width and the respective facial height measurement) for inclusion in the fWHR analyses. PCAs revealed three components should be retained; PC1 represented a length-based allometric component (e.g. height and acromial height) whereas PC2 (e.g. hip breadth and thigh circumference) and PC3 (e.g. forearm circumference, shoulder circumference and jaw size) represented size-based allometric components (e.g. see electronic supplementary material for more details on PCAs) which arguably represent female-biased and male-biased sexual dimorphism, respectively.

**Figure 2.**
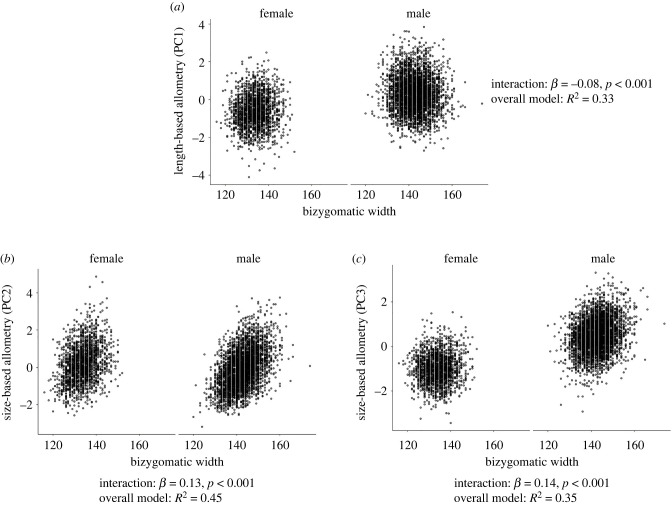
Interactions between allometry and sex on bizygomatic width. Bizygomatic width was measured in millimetres. Darker and lighter shades represent a greater and lesser concentration of participants, respectively. (*a*) Length-based allometry, (*b*) size-based allometry (female-biased sexual dimorphism) and (*c*) size-based allometry (male-biased sexual dimorphism).

We then examined whether the three PCs moderated sexual dimorphism in bizygomatic width (figure 2; darker and lighter shades represent a greater and lesser concentration of participants, respectively), fWHRmenton (electronic supplementary material, figure S1) and fWHRtragion (electronic supplementary material, figure S2). We entered the interaction between sex and each PC (as well as their main effects) in multiple regression models. There were negative interactions between length-based allometry and sex on bizygomatic width and fWHR and positive interactions between size-based allometry on bizygomatic width and fWHR, indicating that allometry affects the sexual dimorphism of bizygomatic width and fWHR. Models involving bizygomatic width as the outcome variable of interest were more accurate; models explaining bizygomatic were up to 15 times more accurate than models explaining fWHR (e.g. for the interaction between sex and PC2 on bizygomatic width, *R*-squared = 0.45; for the interaction between sex and PC2 on fWHRmenton, *R*-squared = 0.03, see electronic supplementary material, figure S1).

### (d) Multiple regression analyses

Given that allometry affects the sexual dimorphism of bizygomatic width and fWHR, in line with previous guidelines [26], we modelled the three allometric PCs as covariates in multiple regressions with sex as a predictor of bizygomatic width, fWHRmenton and fWHRtragion. This revealed pronounced male-biased sexual dimorphism in bizygomatic width, *β =* 0.47, *t*_6063_ = 27.63, *p* < 0.001, equivalent *d* = 1.07, but inconsistent results with fWHR. There were contradictory associations with small effect sizes between sex and fWHRmenton, *β =* −0.12, *t*_6063_ = −5.26, *p* < 0.001, equivalent *d* = −0.24, and fWHRtragion, *β =* 0.08, *t*_6063_ = 3.88, *p* < 0.001, equivalent *d* = 0.16.

## 4. Discussion

fWHR has been arguably the most popular metric in the last 15 years of face perception research, putatively predicting male assertiveness, dominance and aggressiveness [1]. Yet conflicting evidence across studies has challenged the long-accepted view that mid-face robustness is sexually dimorphic [9]. We used the largest dataset to date on facial and bodily morphology to uncover whether sex differences in fWHR are robust when controlling for allometry. After controlling for 92 correlated anatomical measurements, including multiple facial height measurements, we found mixed statistically significant evidence for sex differences in fWHR. Instead, we found pronounced sexual dimorphism in bizygomatic width (*d* = 1.39) that remained after controlling for allometry (*d* = 1.07). We suggest that mixed findings regarding sexual dimorphism in fWHR likely occurred due to relying on discrete ratios [26–28] and not employing multivariate measures to expose facial sexual dimorphisms from among suites of correlated bodily indices [33].

Uncovering the morphological features that share allometric relationships with targets of sexual selection are vital for establishing how competition may have shaped human secondary sexual traits [9,33]. Here, bizygomatic width showed positive allometry with multiple bodily indices, with the strongest associations occurring between bizygomatic width and shoulder breadth, bicep circumference, forearm circumference, chest circumference, neck circumference and body size. These associations between bizygomatic width and surrounding bodily features provide the most compelling support to date for the argument that faces are evolved reliable indicators of body size and formidability [40–44].

To advance the state of this important research area, we echo the recommendations of recent critiques of anatomical ratio measures [26]. While there may be statistical problems with anatomical ratio measurements [26–28], there may also be theoretical problems (see [9]). The original intention behind fWHR was to adjust for allometry [4] but ratio approaches do not adjust for allometry [26,28] and instead create new and specific measurements with unusual properties that may be prone to spurious correlations [28]. Nakagawa *et al*. [26] advised that researchers avoid anatomical ratio measurements for both statistical and theoretical reasons and that, instead of dividing two measurements to adjust for allometry, ‘raw measurements or their direct transformations should be used to control for confounding effects’ [26]. Future research might wish to use univariate measures such as body size and height or, more preferably, multivariate approaches [10–25,43–47]. This includes shape analyses, which often adjust for allometry by scaling units to their centroid size [43–47]. These analyses indeed appear to indicate male-biased sexual dimorphism in facial width [45].

The simple approaches taken in studies of sexual selection among human populations contrasts with those typically employed by evolutionary biologists studying non-human species, where researchers routinely use multivariate frameworks to understand the evolution of suites of correlated traits [4,26]. While a large body of empirical research implicates men's fWHR in dozens of physiological and psychological outcomes [1], the reliance on ratio measurements fails to capture the complex multivariate relationships between measures that define sexually selected traits [2,4,26]. Unfortunately, such approaches may lead to spurious correlations and biased effect-size estimates [26–28], with detrimental effects on the reproducibility of behavioural research. Our findings point to the importance of correct modelling practices and employing multivariate allometric scaling when testing whether human facial morphology is sexually dimorphic, with important implications for studying whether sexual selection has shaped the human face.

## Data Availability

Data are publicly available on the Open Science Framework (https://osf.io/yckrd/). It contains all 93 anatomical measurements present in the paper, sex (0: female, 1: male), birth locations, log transformed, within-group centred and Z-standardized measurements, as well as the principal components and ratio measures used in the analyses. The data are provided in the electronic supplementary material [48].
